# Supplementary material for: One- and Two-Year Effects of the Healthy Primary School of the Future on Children’s Dietary and Physical Activity Behaviours: A Quasi-Experimental Study
Source: Nutrients. 2019 Mar 22;11(3):689. doi: 10.3390/nu11030689 (PMC6470547; doi:10.3390/nu11030689)
Supplement: Supplementary file 1 [file nutrients-11-00689-s001.pdf]

Additional file

Additional table 1: Children’s PA and dietary behaviours per item: descriptives and effect sizes

Total score	Items		N total	Full HPSF		Partial HPSF		Control		Effect size	
				% / Mean (±SD)	% Imp.*	% / Mean (±SD)	% Imp.*	% / Mean (±SD)	% Imp.*	HPSF vs control	PAS vs control
**Parent-reported**											
**Total time spend on PA behaviours**	**Active school transport (min/day)**	T0	740	8.76 (±10.30)		8.68 (±11.33)		11.36 (±14.58)			
		T1	701	8.57 (±9.65)	27.6	9.64 (±10.80)	30.1	12.03 (±13.14)	36.1	-0.07	0.03
		T2	632	10.79 (±11.81)	37.4	9.95 (±14.32)	22.7	12.34 (±20.31)	33.1	0.01	0.00
	**Playing outside after school (min/day)**	T0	734	51.36 (±46.65)		45.88 (±41.90)		50.80 (±45.53)			
		T1	692	46.58 (±43.55)	35.6	45.16 (±38.98)	43.5	54.45 (±49.47)	41.8	-0.14	-0.06
		T2	617	39.84 (±36.19)	33.1	43.36 (±42.40)	39.2	53.29 (±47.69)	43.1	-0.25	-0.03
	**Playing inside active after school (min/day)**	T0	727	36.25 (±43.05)		34.83 (±37.75)		41.94 (±46.87)			
		T1	598	35.62 (±39.78)	38.7	33.95 (±33.59)	44.5	40.57 (±43.07)	43.8	0.01	0.00
		T2	615	28.84 (±30.68)	36.7	29.22 (±31.85)	33.8	39.25 (±44.70)	40.9	-0.06	-0.05
	**Sports club (min/day)**	T0	732	21.21 (±21.33)		18.33 (±17.48)		19.44 (±18.65)			
		T1	695	21.33 (±18.54)	32.9	22.52 (±17.67)	44.1	24.22 (±28.75)	40.3	-0.18	-0.03
		T2	616	23.33 (±19.32)	44.2	24.86 (±19.53)	60.3	26.07 (±24.56)	47.9	-0.10	0.05
**Total time spend on sedentary behaviours**	**Watching TV (min/day)**	T0	735	60.23 (±42.71)		62.98 (±41.09)		62.28 (±40.39)			
		T1	692	55.34 (±36.71)	39.3	63.39 (±37.91)	26.3	63.74 (±40.17)	26.3	-0.22	-0.03
		T2	612	52.83 (±36.84)	42.1	62.03 (±38.72)	30.2	61.21 (±41.36)	29.8	-0.22	-0.05
	**Using computer (min/day)**	T0	735	29.71 (±38.08)		26.65 (±35.92)		32.08 (±39.24)			
		T1	689	28.20 (±31.56)	36.4	28.88 (±36.86)	28.3	32.93 (±40.11)	28.9	-0.15	-0.02
		T2	611	28.11 (±33.93)	31.4	26.80 (±31.96)	28.9	34.86 (±39.60)	27.8	-0.23	-0.17
	**Social media (min/day)**	T0	726	1.53 (±10.74)		1.65 (±8.02)		3.92 (±21.40)			
		T1	690	4.30 (±19.81)	4.9	3.33 (±13.43)	3.7	5.48 (±20.83)	5.9	0.05	0.03
		T2	604	3.46 (±11.92)	3.3	5.76 (±19.97)	1.6	5.96 (±19.58)	2.6	-0.04	0.03
**Children’s healthy dietary behaviours**	**Breakfast (days/week)**	T0	717	6.79 (±0.80)		6.85 (±0.76)		6.82 (±0.81)			
		T1	688	6.85 (±0.75)	3.1	6.81 (±0.87)	2.4	6.76 (±1.00)	2.0	0.12	0.07
		T2	602	6.87 (±0.77)	5.0	6.87 (±0.85)	3.2	6.75 (±1.01)	1.8	0.19	0.10
	**Fruit (days/week)**	T0	715	5.57 (±1.59)		5.40 (±1.66)		4.99 (±1.86)			
		T1	685	5.70 (±1.52)	28.1	5.47 (±1.81)	22.8	4.95 (±1.91)	25.3	0.12	0.09
		T2	593	5.60 (±1.51)	21.0	5.36 (±1.60)	28.2	4.98 (±1.87)	26.4	-0.03	-0.03
	**Vegetables raw (days/week)**	T0	715	3.67 (±2.22)		3.53 (±2.18)		3.36 (±2.14)			
		T1	685	4.13 (±2.14)	48.1	3.97 (±2.04)	40.7	3.73 (±2.17)	38.0	0.06	-0.03
		T2	593	4.16 (±2.12)	47.1	4.33 (±1.98)	46.8	3.86 (±2.16)	49.1	-0.01	0.04
		T0	715	4.82 (±1.60)		4.95 (±1.57)		4.93 (±1.53)			

	**Vegetables cooked/baked (days/week)**	T1	685	4.91 (±1.61)	31.3	4.93 (±1.62)	28.7	4.87 (±1.42)	28.7	0.07	0.04
		T2	593	4.94 (±1.59)	39.5	4.72 (±1.56)	30.6	4.91 (±1.59)	32.1	0.06	-0.11
	**Water (days/week)**	T0	711	4.83 (±2.66)		5.16 (±2.35)		4.61 (±2.77)			
		T1	684	5.64 (±2.04)	34.4	5.81 (±2.07)	28.3	4.97 (±2.60)	26.0	0.21	0.10
		T2	590	6.03 (±1.64)	38.7	5.52 (±2.19)	30.9	5.10 (±2.49)	29.2	0.36	0.02
**Children's unhealthy dietary behaviours**	**Soft drinks (days/week)**	T0	711	3.76 (±2.53)		3.83 (±2.59)		4.07 (±2.63)			
		T1	684	3.01 (±2.60)	48.8	3.30 (±2.73)	42.8	3.73 (±2.70)	34.0	-0.19	-0.10
		T2	590	2.95 (±2.52)	44.5	3.48 (±2.70)	41.5	3.31 (±2.54)	40.6	-0.11	0.06
	**Sport drinks (days/week)**	T0	711	0.09 (±0.38)		0.09 (±0.37)		0.12 (±0.48)			
		T1	684	0.08 (±0.38)	.6	0.09 (±0.37)	.0	0.23 (±0.67)	.7	-0.16	-0.21
		T2	590	0.09 (±0.35)	.8	0.10 (±0.52)	.0	0.13 (±0.48)	.0	0.02	0.00
	**Energy drinks (days/week) [a]**	T0	711	0.01 (±0.13)		0.00 (±0.00)		0.01 (±0.14)			
		T1	684	0.00 (±0.00)	3.8	0.00 (±0.00)	6.0	0.02 (±0.27)	2.0		
		T2	589	0.00 (±0.00)	4.2	0.00 (±0.00)	4.9	0.00 (±0.07)	4.7		
	**Chocolate (days/week)**	T0	704	0.83 (±1.45)		0.76 (±1.46)		0.95 (±1.44)			
		T1	679	0.75 (±1.36)	21.3	0.77 (±1.44)	20.1	1.06 (±1.48)	20.1	-0.18	-0.05
		T2	586	0.97 (±1.56)	21.8	0.96 (±1.46)	17.9	1.14 (±1.48)	16.5	-0.09	0.00
	**Salted snacks (days/week)**	T0	704	1.33 (±1.28)		1.24 (±1.04)		1.30 (±1.24)			
		T1	679	1.36 (±1.13)	25.0	1.29 (±1.24)	21.3	1.53 (±1.31)	19.5	-0.14	-0.13
		T2	586	1.44 (±1.40)	24.4	1.38 (±1.16)	22.8	1.41 (±1.37)	23.3	-0.01	0.00
	**Cookies (days/week)**	T0	704	0.54 (±0.99)		0.43 (±0.80)		0.73 (±1.30)			
		T1	679	0.53 (±0.91)	23.1	0.41 (±0.80)	20.7	0.58 (±0.96)	22.1	0.17	0.14
		T2	586	0.54 (±1.03)	19.3	0.51 (±1.01) [c]	19.5	0.51 (±0.79)	24.3	0.24	0.29
	**Soft ice-cream (days/week)**	T0	704	1.06 (±1.35)		1.02 (±1.53)		1.08 (±1.35)			
		T1	679	1.08 (±1.43)	32.5	1.12 (±1.36)	28.7	1.20 (±1.48)	28.2	-0.15	-0.08
		T2	586	1.12 (±1.33)	34.5	1.08 (±1.36)	27.6	1.40 (±1.87)	22.3	-0.23	-0.26
**Child-reported**											
**Children's healthy dietary behaviours**	**Daily breakfast (% yes) [b]**	T0	778	90.7		90.1		90.0			
		T1	1015	93.3	5.0	87.6	2.8	85.9	3.8	2.42	1.16
		T2	1051	90.5	4.2	88.9	.7	84.1	4.9	1.76	1.30
	**Fruit intake (0-3) [c]**	T0	745	2.18 (±0.89)		2.07 (±0.93)		2.01 (±0.83)			
		T1	993	2.09 (±0.86)	22.4	2.14 (±0.89)	25.4	1.94 (±0.88)	20.1	-0.03	0.14
		T2	1027	2.02 (±0.86)	24.8	2.06 (±0.93)	30.8	1.99 (±0.87)	23.5	-0.16	0.03
	**Vegetables intake (0-3) [c]**	T0	687	2.22 (±0.85)		2.12 (±0.86)		2.14 (±0.76)			
		T1	940	2.13 (±0.87)	21.6	2.20 (±0.81)	25.3	2.12 (±0.76)	20.8	-0.07	0.12
		T2	962	2.21 (±0.85)	35.0	2.00 (±0.89)	35.1	2.08 (±0.82)	33.8	0.04	-0.05
	**Water consumption (0-3) [c]**	T0	774	2.07 (±0.92)		2.05 (±1.01)		2.02 (±0.92)			
		T1	1015	2.10 (±0.97)	29.1	2.07 (±1.02)	25.0	2.13 (±0.98)	28.0	-0.07	-0.12
		T2	1045	2.14 (±1.00)	35.2	2.15 (±0.98)	32.4	2.08 (±1.04)	31.7	0.01	0.03
	**Soft drinks consumption (0-3) [c]**	T0	763	1.90 (±0.99)		1.84 (±1.00)		1.92 (±0.92)			
		T1	1006	1.69 (±0.94)	34.7	1.48 (±1.01)	44.9	1.70 (±0.96)	35.1	0.02	-0.16

		T2	1044	1.64 (±1.00)	45.3	1.54 (±0.99)	46.7	1.57 (±1.02)	43.7	0.10	0.01
**Children's unhealthy dietary behaviours**	**Sport drinks consumption (0-3)** [c]	T0	774	0.96 (±0.98)		0.81 (±0.92)		0.91 (±0.93)			
		T1	1015	0.64 (±0.82)	36.6	0.58 (±0.85)	32.1	0.67 (±0.84)	35.5	-0.07	-0.01
		T2	1047	0.59 (±0.89)	42.6	0.50 (±0.78)	42.4	0.66 (±0.89)	40.5	-0.12	-0.13
	**Energy drinks consumption (0-3)** [c]	T0	771	0.27 (±0.64)		0.38 (±0.80)		0.28 (±0.69)			
		T1	1011	0.23 (±0.59)	10.9	0.15 (±0.49)	18.0	0.14 (±0.43)	14.6	0.15	-0.25
		T2	1045	0.25 (±0.68)	14.1	0.13 (±0.48)	22.9	0.23 (±0.64)	14.2	-0.02	-0.50
	**Chocolate intake (0-3)** [c]	T0	776	1.26 (±0.90)		1.19 (±0.92)		1.30 (±0.86)			
		T1	1013	1.17 (±0.94)	31.9	0.95 (±0.95)	41.5	1.07 (±0.91)	37.4	0.19	-0.07
		T2	1045	1.27 (±0.95)	32.2	0.98 (±0.92)	55.0	1.08 (±0.94)	43.0	0.24	-0.13
	**Salted snacks intake (0-3)** [c]	T0	772	1.18 (±0.83)		1.15 (±0.86)		1.21 (±0.81)			
		T1	1019	1.07 (±0.89)	34.0	0.92 (±0.81)	40.1	0.99 (±0.82)	37.1	0.15	-0.02
		T2	1046	1.07 (±0.91)	40.1	0.89 (±0.82)	54.3	1.10 (±0.89)	41.0	0.00	-0.27
	**Cookies intake (0-3)** [c]	T0	773	0.99 (±0.76)		0.97 (±0.82)		1.09 (±0.79)			
		T1	1019	0.80 (±0.84)	38.2	0.67 (±0.78)	42.1	0.77 (±0.79)	45.3	0.18	0.01
		T2	1045	0.86 (±0.85)	47.9	0.68 (±0.81)	53.2	0.81 (±0.86)	55.0	0.21	-0.05
	**Soft ice-cream intake (0-3)** [c]	T0	776	1.35 (±0.85)		1.42 (±0.91)		1.49 (±0.86)			
		T1	1017	1.40 (±0.97)	31.1	1.21 (±0.91)	41.9	1.31 (±0.92)	38.4	0.27	-0.06
		T2	1045	1.34 (±0.91)	36.1	1.14 (±0.91)	50.7	1.30 (±0.95)	42.1	0.22	-0.16

* Improved: Percentage of children who improved, i.e., changed in a favorable direction, compared with their baseline score.
[a] No variation in variable; effect sizes could not be conducted.
[b] Effect size is odds ratio.
[c] Range: 0 (never) to 3 (every day).

Additional table 2: Lunch food type combinations

		N total	Full HPSF %	Full HPSF % Imp.*	Partial HPSF %	Partial HPSF % Imp.*	Control %	Control % Imp.*	Effect size: Full HPSF vs control	Effect size: Partial HPSF vs control
**Child-reported**										
**Grains [a] and fruit (% yes)**	T0	854	35.2		35.3		25.9			
	T1	1201	57.9	37.1	31.8	12.0	27.9	15.5	2.31	0.75
	T2	1143	49.6	30.2	39.0	20.2	31.3	21.6	1.37	0.85
**Grains and vegetables (% yes)**	T0	853	23.2		23.0		14.2			
	T1	1201	49.4	41.6	17.5	8.1	18.6	13.4	2.48	0.49
	T2	1138	60.2	44.2	19.1	13.9	20.8	17.4	3.57	0.50
**Dairy [b] and fruit (% yes)**	T0	841	14.2		18.6		12.4			
	T1	1193	46.5	38.7	13.3	8.6	15.9	12.0	4.00	0.47
	T2	1144	40.1	27.5	13.8	9.6	16.2	14.6	2.98	0.48
**Dairy and vegetables (% yes)**	T0	845	10.7		11.4		8.7			
	T1	1197	38.5	34.0	7.2	4.7	10.6	8.4	4.31	0.47
	T2	1144	47.1	39.6	7.7	5.9	11.5	8.7	5.75	0.46
**Grains and dairy (% yes)**	T0	845	34.4		38.1		32.2			
	T1	1199	69.5	40.5	28.9	11.6	37.1	21.3	3.34	0.51
	T2	1139	68.0	41.3	25.8	10.8	37.1	23.4	3.14	0.42

* Improved: Percentage of children who improved, i.e., changed in a favorable direction, compared with their baseline score.

[a] Grains: bread and cereals

[b] Dairy: milk, yoghurt, and cheese
